# Revolutionizing Spinal Care: Current Applications and Future Directions of Artificial Intelligence and Machine Learning

**DOI:** 10.3390/jcm12134188

**Published:** 2023-06-21

**Authors:** Mitsuru Yagi, Kento Yamanouchi, Naruhito Fujita, Haruki Funao, Shigeto Ebata

**Affiliations:** 1 Department of Orthopaedic Surgery, School of Medicine, International University of Health and Welfare, Narita 286-8686, Japan; yamaken0331@gmail.com (K.Y.); naruhito88@hotmail.com (N.F.); hfunao@yahoo.co.jp (H.F.); 2 Department of Orthopaedic Surgery, International University of Health and Welfare and Narita Hospital, Narita 286-8520, Japan; ebatas310@gmail.com

**Keywords:** artificial intelligence, machine learning, predictive model

## Abstract

Artificial intelligence (AI) and machine learning (ML) are rapidly becoming integral components of modern healthcare, offering new avenues for diagnosis, treatment, and outcome prediction. This review explores their current applications and potential future in the field of spinal care. From enhancing imaging techniques to predicting patient outcomes, AI and ML are revolutionizing the way we approach spinal diseases. AI and ML have significantly improved spinal imaging by augmenting detection and classification capabilities, thereby boosting diagnostic accuracy. Predictive models have also been developed to guide treatment plans and foresee patient outcomes, driving a shift towards more personalized care. Looking towards the future, we envision AI and ML further ingraining themselves in spinal care with the development of algorithms capable of deciphering complex spinal pathologies to aid decision making. Despite the promise these technologies hold, their integration into clinical practice is not without challenges. Data quality, integration hurdles, data security, and ethical considerations are some of the key areas that need to be addressed for their successful and responsible implementation. In conclusion, AI and ML represent potent tools for transforming spinal care. Thoughtful and balanced integration of these technologies, guided by ethical considerations, can lead to significant advancements, ushering in an era of more personalized, effective, and efficient healthcare.

## 1. Introduction

The management of spinal diseases is on the cusp of a transformative shift precipitated by the emergence and integration of artificial intelligence (AI) and machine learning (ML) into the realm of standard medical care [1,2,3,4,5,6]. Rather than being a vision of the distant future, this shift towards an intelligence-based spinal care model is well underway, promising a host of potential applications, including diagnosis, treatment, and the anticipation of adverse events [1,2,3,4,5,6].

The advent of AI and ML in healthcare is not an isolated phenomenon but rather the logical outcome of decades of accumulated scientific and technological progress within computational and healthcare disciplines. AI and ML have transcended mere theoretical promise; they are already delivering tangible results in the present day [1,2,3,4,5,6]. One of the most striking examples of their efficacy lies in the realm of spinal imaging [7]. Sophisticated algorithms augment the creation and interpretation of spinal images, thereby enriching the decision-making data available to clinicians [7]. It is plausible that future radiologists will collaborate seamlessly with these AI-driven systems to deliver more precise and personalized care.

This paper aims to provide a comprehensive review of the current state and projected advancements in AI and ML applications in spinal disease management. It further endeavors to elucidate the potential hurdles that may be encountered in the integration of these avant-garde technologies into routine clinical practice. It will also address the critical ethical and regulatory considerations tied to the deployment of AI and ML in healthcare. As we traverse this path of rapid technological evolution, it becomes paramount to assess our progress continually and to chart a course for the future that balances knowledge, ethical responsibility, and, above all, the welfare of patients.

## 2. Historical Context and Evolution

The advent of AI has irrevocably changed numerous fields, and healthcare is no exception [1,2,3,4,5,6]. AI—broadly defined as the ability of a computer or computer-controlled robot to perform tasks commonly associated with intelligent beings—has introduced a level of sophistication in data analysis and decision making that was previously unattainable.

The roots of AI in healthcare are intertwined with the history of AI itself (Table 1). The initial AI wave in the mid-20th century saw promising applications in healthcare, but many of these early attempts were hampered by the limited computational power and data availability of the time [1,2,3,4,5,6,8]. However, the situation has drastically changed. The past few decades have witnessed the exponential growth of computing power and data generation, thanks to the advent of the digital era, leading to the explosive growth of AI capabilities. This growth is encapsulated in the “scaling hypothesis”, which posits that increases in the size of neural networks, the number of training data, and computation, have led to astonishing advances in AI.

A key factor in this growth is ML, a subset of AI that focuses on the development of algorithms that enable computers to learn from and make decisions based on data [8]. In healthcare, ML has become a critical tool for predictive analytics, personalized medicine, and disease diagnosis and prognosis.

One significant application of ML in healthcare is the use of artificial neural networks (ANNs)—computational models that emulate the human brain’s structure and function. ANNs consist of layers of interconnected “neurons” or nodes that transmit and process information [9]. Their remarkable ability to learn from data without being explicitly programmed to perform specific tasks makes them suitable for a wide array of applications, including medical diagnosis, drug discovery, and patient care management [9,10,11].

In the context of spinal disease management, ANNs have begun to find their footing. The last few decades have seen a growing body of research exploring their potential in diagnosing spinal diseases, predicting treatment outcomes, and even forecasting the likelihood of adverse events [9,10,11]. These developments are part of a larger shift towards data-driven, personalized, and predictive healthcare that promises to significantly improve patient outcomes and the efficiency of the healthcare system.

Thus, the incorporation of AI, ML, and particularly ANNs in healthcare represents an important evolution, ushering in a new era in spinal disease management. The scale of this revolution and its potential impact can hardly be overstated. However, it is vital to approach these technologies with an understanding of their strengths, limitations, and the ethical considerations that accompany their use to ensure that their integration into clinical practice is thoughtful, equitable, and ultimately beneficial for all patients.

## 3. Current Applications of AI and ML in Spinal Care

AI and ML have seen significant developments and implementations in recent years, particularly in the domain of healthcare (Table 2). Spinal care—a critical aspect of the healthcare system—has been no exception to this trend. Over time, these technologies have been utilized in various capacities in the sphere of spinal care, ranging from disease diagnosis to treatment and even the prediction of adverse events (Table 3) [8,12,13,14,15].

## 4. Clinical Case Studies: AI in Spinal Care

AI and ML have gained significant traction in spinal disease diagnosis, treatment recommendation, and patient outcome prediction. One notable case study is the work of Jujjavarapu et al., who used a deep-learning model to predict surgical outcomes in patients with lumbar disc herniation and lumbar spinal stenosis [16]. The study demonstrated that the AI model outperformed a benchmark model (logistic regression) in predicting early surgery, achieving an AUC of 0.725 compared to 0.597.

Another compelling case study was presented by Halicka et al., who developed an AI algorithm capable of predicting patient-reported outcomes following lumbar spine surgery [17]. The study aimed to develop and externally validate prediction models for spinal surgery outcomes using multivariate regression and random-forest approaches. The study included patients who underwent lumbar spine surgery for degenerative pathology. The models were evaluated based on changes in back and leg pain intensity and Core Outcome Measures Index (COMI) scores. The models demonstrated good calibration and explained variations in the validation data. The discrimination ability ranged from 0.62 to 0.72, indicating moderate predictive performance. The most important predictors included age, baseline scores, type of degenerative pathology, previous surgeries, smoking status, morbidity, and hospital-stay duration. The developed models showed robustness but had borderline-acceptable discrimination abilities, suggesting the need for additional prognostic factors.

In addition to its diagnostic applications, AI has shown promise in predicting hospital stays following spine surgery. Shahrestani et al. conducted a study in which algorithms were trained using preoperative and perioperative variables from a dataset of patients with spondylolisthesis [18]. In the study conducted by Shahrestani et al., the researchers aimed to develop k-nearest-neighbors (KNN) classification algorithms to identify patients at a higher risk of extended hospital length of stay (LOS) following spinal surgery for spondylolisthesis. They analyzed the Quality Outcomes Database (QOD) spondylolisthesis dataset, including preoperative and perioperative variables. Out of 608 enrolled patients, 544 met the inclusion criteria. The KNN models exhibited impressive predictive performance. Model 1 achieved an overall accuracy of 98.1%, a sensitivity of 100%, a specificity of 84.6%, a positive predictive value (PPV) of 97.9%, and a negative predictive value (NPV) of 100%. Model 2 demonstrated an overall accuracy of 99.1%, a sensitivity of 100%, a specificity of 92.3%, a PPV of 99.0%, and an NPV of 100%. Receiver operating characteristic (ROC) curve analysis revealed an area under the curve (AUC) of 0.998 for both models. The study concluded that these nonlinear KNN machine-learning models have exceptional predictive value for LOS and can potentially assist in patient selection, management, resource utilization, and preoperative surgical planning. These models have the potential to assist spine surgeons in patient selection, the optimization of resource utilization, and preoperative planning.

These case studies serve as tangible examples of the benefits that AI and ML can bring to spinal care. They highlight that AI is not merely a futuristic concept but a current tool that is being utilized to enhance patient care. However, it is important to note that the application of these technologies is still in its early stages, and further research and clinical trials are needed to refine these tools and fully unlock their potential.

The introduction of AI and ML in spinal care signifies a paradigm shift toward an AI-augmented care model. An understanding of the evolution of computation in this context is crucial to appreciate the potential impact on diagnosis, treatment, and adverse-event prediction.

Decision-tree models have been used in predicting hospital readmission, prolonged hospital say, surgical complication, and direct cost following surgery for spinal stenosis with a high degree of accuracy [19,20,21,22]. They have also been utilized for texture analysis of spinal stenosis from MR imaging.

On another front, ANNs have been deployed to predict non-home discharge after spinal stenosis surgery [23]. Other models have predicted patient outcomes, including pain and functional disability after spinal stenosis surgery [19,20,21,22,24]. As the research evolves, future iterations of these AI and ML models could incorporate additional patient features, such as body mass index, lean mass, or gender, to further improve diagnostic and prognostic capabilities.

Natural language processing (NLP)—another application of AI—has also been explored in the context of spinal care [25,26]. For instance, an NLP system was developed and found to have a higher sensitivity for identifying standard reporting characteristics for low back pain on radiologic imaging compared to its rule-based counterpart [25]. This suggests that future NLP systems using ML could potentially enhance pathology-specific word choice to refine diagnosis and treatment strategies.

In addition to the more traditional ML techniques, support vector machines (SVMs) have also been used to classify patients with low back pain based on progression following rehabilitation [27]. A model developed by Jiang et al. achieved a striking 100% sensitivity and a 93.75% accuracy, hinting at the possibility of preoperative identification of patients who may require additional or more intensive rehabilitation efforts [27].

Comparatively, ANNs have been tested against gold-standard diagnostic categorization of low back pain in patients. The results revealed a high sensitivity and specificity of 95.7% and 100%, respectively. The successful combination of these ML algorithms with additional diagnostic tests could potentially revolutionize the clinician’s diagnostic process [28].

Predictive models have also been employed to identify the risk of major intraoperative or perioperative complications following adult spinal deformity [19,22,29,30,31]. Using preoperative variables, including coronal and sagittal radiographic images, a decision-tree model was developed by Scheer et al. and Yagi et al. [19,22]. This kind of risk assessment tool can provide invaluable assistance in surgical planning and patient counseling.

In summary, AI and ML have brought a transformative change to spinal care, offering significant advancements in disease detection, patient outcome prediction, risk assessment, and therapeutic decision making. The above applications demonstrate just a fraction of their potential, and ongoing research in this field promises further breakthroughs that will revolutionize the field of spinal care. The future of spinal care may be one in which AI and ML are integral to all stages of care, from diagnosis to treatment and prognosis, providing physicians with tools to offer more personalized and effective care.

## 5. AI and ML in Spinal Imaging

Spinal imaging is a cornerstone of the diagnosis and management of various spinal disorders. Traditionally, these images have been analyzed manually by clinicians—a process that can be time-consuming and susceptible to human error. However, the advent of AI and ML has transformed this situation, enabling automated, fast, and precise image interpretation.

One of the fundamental applications of AI in spinal care revolves around localization—a concept associated with object detection and classification [32,33,34,35,36]. This concept enables the identification and labeling of an object in an image and can be invaluable in detecting anomalies in the spine. The SVM represents one such ML model that has demonstrated effectiveness in this field. SVMs have been used in detecting incidental lumbar spine fractures on X-rays, predicting forces applied to the lower back during weighted loading, and even characterizing type 1 Gaucher disease based on bone microarchitecture [37,38].

Similarly, the random-forest model, another ML technique, has been employed to identify osteoporosis more effectively than traditional bone turnover markers alone. It has also been used in the screening of patients undergoing non-osteoporosis dedicated CT imaging for potential osteoporosis [39].

Neural networks have found use in predicting fractures, both of the spine and the hip. Notably, these networks have been trained to detect posterior-element spinal fractures in trauma patients using CT images. Further, they have been used to identify hip fractures using a combination of radiographs, patient traits, and hospital process variables. In an exciting development, researchers found that image recognition algorithms using both imaging and non-imaging data may primarily use non-imaging data [40].

Convoluted neural networks (CNNs)—an offshoot of neural networks—have been developed to characterize and classify alignment-related pathologies, such as kyphosis and scoliosis [41,42]. One such CNN generated by Jamaludin et al. from dual-energy X-ray absorptiometry (DEXA) scans was able to automate spine curve identification, boasting a sensitivity of 86.5%, a specificity of 96.9%, and an AUC (area under the ROC curve) of 0.80 [43]. This capability opens the possibility for earlier detection of alignment-related pathologies, such as scoliosis and kyphosis.

Regression techniques have also been incorporated in ML for spinal care, with logistic regression models predicting the development of neuromuscular scoliosis in pediatric patients with cerebral palsy. Linear regression models have been used for postoperative height gain following the correction of idiopathic scoliosis [44].

Clustering methods have been used to identify distinct subgroups within adolescent idiopathic scoliosis populations. However, it has been challenging to identify discriminatory characteristics for patient clustering in certain study sets [42].

AI has also been beneficial in diagnosing various types of spinal pathologies, including lumbar neural foraminal stenosis and central spinal stenosis [10,15,44,45]. Deep neural networks have been employed to automatically localize and grade multiple spinal regions. These ML methods have the potential to reduce the qualitative MRI grading time in large epidemiological studies. Similarly, deep neural networks have been utilized to automatically localize and grade multiple spinal regions to diagnose conditions such as lumbar neural foraminal stenosis [44,45,46].

In another study, Roller et al. applied CNNs to MRI images to predict the operative level of patients undergoing disc decompression surgery [36]. An algorithm has also been developed to predict patients at risk for re-herniation after microdiscectomy, achieving a recall of 0.80 and an accuracy of 0.70 [36].

AI has even been used to predict early-onset adjacent segment degeneration following anterior cervical discectomy fusion (ACDF) using an SVM on tabular data [47].

Overall, AI and ML techniques in spinal imaging have shown promising results in improving the accuracy, speed, and predictive capability of the diagnosis and treatment of spinal conditions. These developments hold great promise for improving patient outcomes and transforming the way spinal care is delivered. However, it is essential to continue to refine these AI and ML models, incorporating new insights and additional patient features to ensure their continued evolution and relevance in clinical practice.

## 6. Role of AI and ML in Spinal Rehabilitation

AI and ML have diverse applications in rehabilitation, including applications in the field of physical medicine and rehabilitation (PM&R). In rehabilitation, ML is utilized in various areas, including symbiotic neuroprosthetics, myoelectric control, brain–computer interfaces, perioperative medicine, musculoskeletal medicine, diagnostic imaging, patient data measurement, and clinical decision support [48,49,50]. AI has even been used to assess rehabilitative exercises based on machine indications [48,49,50,51]. Brain–computer interfaces (BCIs) have emerged as a novel approach in neurorehabilitation. By recording and decoding brain signals, BCIs aim to enhance motor imagery-based training, facilitate task execution through functional electrical stimulation or robotic orthoses, and understand cerebral reorganizations after injury. BCIs show potential in promoting recovery and can be adapted to a diverse population. However, controlled clinical trials are needed to validate their effectiveness in pathological conditions and compare them to traditional methods [48,49]. Recently, Simmonov et al. presented an innovative rehabilitation strategy utilizing AI [51]. They explored the use of humanoid robots in pulmonary rehabilitation for COPD patients. The Aldebaran Robotics’ NAO humanoid was programmed to assist patients during rehabilitation exercises and assess their performance using the dynamic time warping (DTW) algorithm. The study highlights the potential of intelligent humanoid applications to enhance rehabilitative care and minimize the reliance on human intervention.

As PM&R providers, we have the opportunity to harness these AI and ML tools to advance patient care and contribute to the progress of the field.

## 7. Validity and Reliability: Ensuring Accuracy in AI-Driven Diagnosis and Treatment

The promise of AI and ML in spinal care is underpinned by their ability to accurately and reliably assist in diagnosing and treating spinal diseases. However, the validity and reliability of these AI-driven systems are not guaranteed. They need to be thoroughly evaluated and confirmed through rigorous testing, validation, and continuous assessment to ensure the accuracy and reliability of their outputs (Table 4) [52].

AI and ML models are as good as the data they are trained on. Thus, the quality and diversity of the data used play a significant role in the validity of the AI model. If the training data are not representative of the broader population or the specific patient groups, the AI model may perform poorly when deployed in real-world settings. Therefore, using high-quality, diverse, and representative datasets during model training is essential for ensuring the validity of AI models [53].

The reliability of an AI system refers to its ability to consistently produce the same results under the same conditions. This is particularly important in healthcare settings where reliable predictions are crucial for clinical decision making. Variability in AI system performance can lead to different diagnoses or treatment plans for the same patient, which can have serious implications for patient care [54]. Ensuring the validity and reliability of AI systems in spinal care also involves external validation, where the performance of AI models is assessed using data that were not involved in the model’s training or initial validation. This is a crucial step to gauge the generalizability of AI models and their readiness for real-world clinical deployment [55].

Lastly, a system for continuous monitoring and evaluation should be in place. This allows for the detection of any changes in the performance of AI models over time, providing an opportunity to make necessary adjustments and updates [56].

In conclusion, the integration of AI and ML into spinal care promises many benefits, but it is incumbent upon us to ensure the accuracy and reliability of these AI-driven systems. As we navigate this new terrain, the focus must remain on delivering the highest quality of care to patients.

## 8. Future Direction of AI and ML in Spinal Care

The future of spinal care will likely be heavily influenced by advancements in AI and ML. These technologies are poised to redefine the diagnosis, treatment, and overall management of spinal conditions, enhancing precision, speed, and patient outcomes.

While the current applications of AI and ML in spinal care are impressive, the potential for future advancements is enormous. In the realm of diagnosis, we can expect to see AI and ML models that can accurately interpret complex spinal images, detect subtle patterns invisible to the human eye, and even predict the likelihood of certain spinal conditions based on a wide array of factors. For instance, deep-learning algorithms could be refined to not only detect fractures and pathologies but also predict their progression. Similarly, predictive models could be developed to anticipate complications, readmissions, and patient outcomes post-surgery with greater precision.

Treatment strategies may also be revolutionized by AI and ML. Personalized medicine—a treatment approach that tailors therapy to individual patients based on their unique genetic, environmental, and lifestyle factors—could become standard in spinal care. AI and ML could facilitate this by analyzing vast quantities of data to identify the most effective treatment strategies for each patient. This could range from determining the optimal surgical approach to predicting the success of various rehabilitation strategies.

AI and ML could also enhance patient monitoring and follow-up care. Wearable technology and remote monitoring devices could collect real-time data on patients’ health status and recovery, which could then be analyzed using AI algorithms to detect complications or deviations from the expected recovery trajectory [57,58]. This could enable timely intervention, reducing the risk of adverse events and improving patient outcomes.

In addition, AI and ML could play a crucial role in healthcare administration within spinal care. Advanced algorithms could streamline administrative tasks, such as scheduling, billing, and record-keeping, reducing the administrative burden on healthcare providers and allowing them to focus more on patient care [59]. Moreover, AI and ML could be used to optimize resource allocation, ensuring that resources are directed to where they are most needed.

Furthermore, the fusion of AI and ML with other innovative technologies, such as virtual reality and augmented reality, could open new horizons in spinal care. For example, these combined technologies could be used for pre-operative planning and simulation, improving surgical precision and patient outcomes [60,61]. However, to fully realize these potential advancements, several challenges must be addressed. These include ensuring the privacy and security of patient data, managing the vast numbers of data generated by AI and ML algorithms, and ensuring that these technologies are accessible and affordable for all patients. Furthermore, it will be crucial to ensure that healthcare providers are adequately trained in the use of these technologies and that ethical considerations are appropriately addressed.

AI and ML hold immense promise for the future of spinal care. As these technologies continue to advance, they have the potential to transform the way spinal conditions are diagnosed, treated, and managed, ultimately leading to better patient outcomes and more efficient healthcare delivery.

## 9. Addressing Ethical and Regulatory Challenges in AI-Driven Spinal Care

AI and ML continue to make significant strides in spinal care. However, as these technologies become more deeply integrated into our healthcare systems, it is of the utmost importance to address the ethical considerations and understand the evolving regulatory landscape that governs their use [62].

From an ethical standpoint, the advent of AI and ML in healthcare introduces numerous questions. One such critical concern is patient data privacy. Machine-learning models require large numbers of data to function optimally, necessitating stringent measures to ensure that the data collection and processing respect patients’ privacy rights and comply with established data protection laws [63].

Additionally, the deployment of AI and ML in healthcare raises questions about responsibility and accountability. In instances where an AI-driven diagnosis or treatment recommendation proves erroneous, it is essential to ascertain who bears the responsibility—is it the healthcare provider, the developers of the algorithm, or the institution implementing it? This “black box” problem emphasizes the need for transparency and explainability in AI systems [64].

Moreover, there are concerns about data quality and bias. AI and ML models are only as reliable as the data they are trained on. Inaccurate, incomplete, or biased data could lead to flawed models and misguided clinical decisions. This concern underscores the critical need for transparent and interpretable AI and ML models [65].

The ethical considerations also extend to the potential for AI and ML to replace human expertise. While these technologies can augment the abilities of clinicians, it is essential to maintain a balanced approach that leverages the strengths of both human expertise and AI [66]. These technologies should be seen as tools to assist clinicians, not replace them. Furthermore, considering the inherent uncertainties in AI and ML predictions, how can informed consent be ensured when utilizing these technologies in patient care? Patients have a right to comprehend the basis of their treatment decisions, which can be challenging when these decisions are influenced by complex algorithms [67].

Simultaneously, we need to address the regulatory landscape of AI and ML in healthcare, which is currently in its early stages. Bodies such as the U.S. Food and Drug Administration (FDA) and the European Medicines Agency (EMA) have initiated outlining frameworks for regulating AI in healthcare, focusing on safety, effectiveness, and data privacy [68]. However, the swift pace of AI and ML development poses challenges to maintaining current and effective regulatory oversight [69].

In conclusion, while AI and ML hold significant promise for transforming spinal care, their integration into clinical practice must be carried out thoughtfully, keeping in mind the ethical considerations, and in alignment with regulatory requirements [62]. Navigating this evolving landscape necessitates implementing these technologies in ways that benefit all patients, uphold their privacy, and maintain the highest standards of care [70]. As we move forward, addressing these challenges and ethical considerations is paramount to ensure the responsible and effective use of these powerful tools in spinal care [71].

## 10. Conclusions

AI and ML have demonstrated their potential to revolutionize spinal care, offering advancements in diagnosis, treatment, and outcome prediction. The capacity of these technologies to process vast numbers of data, uncover patterns, and learn from experience opens up new opportunities for precision medicine and personalized patient care.

In imaging, AI and ML have been shown to improve the detection and classification of various spinal conditions, offering the potential to enhance diagnostic accuracy and speed. Furthermore, with their predictive capabilities, these technologies may aid in treatment planning and predicting patient outcomes, enhancing the overall quality of care.

Looking ahead, we foresee a future in which AI and ML play an integral role in spinal care. The ongoing advancements in these technologies suggest a future with more precise diagnoses, targeted treatments, and improved patient outcomes. The development of algorithms capable of understanding complex spinal pathologies holds promise for better decision making and improved patient care. However, the journey toward this future is not without challenges. Ensuring data quality, overcoming integration barriers, managing data security, and addressing ethical considerations are all crucial to the successful and responsible application of AI and ML in spinal care.

In summary, while the incorporation of AI and ML in spinal care presents substantial potential benefits, it necessitates a thoughtful and measured approach. A balanced and ethical integration of these technologies can lead to significant advancements in spinal care, shaping a future in which healthcare is more personalized, effective, and efficient.

## Figures and Tables

**Table 1:** Chronological Roots of AI in Healthcare.

Year	Root	Summary
1950s	Expert Systems	Expert systems were among the earliest roots of AI in healthcare. These systems aimed to capture the knowledge and expertise of human experts in specific domains, including healthcare. By codifying expert knowledge into rules and algorithms, expert systems could provide diagnostic and decision-making support, aiding healthcare professionals in making accurate and timely assessments and recommendations.
1960s	Machine Learning	Machine learning emerged as a foundational root of AI in healthcare in the 1960s. Machine-learning algorithms enabled computers to learn from data and improve their performance without explicit programming. In healthcare, machine-learning techniques have been used for tasks, such as pattern recognition, classification, and prediction. Machine-learning models can analyze large volumes of patient data and extract valuable insights, contributing to personalized medicine and clinical decision making.
1970s	Natural Language Processing	Natural language processing (NLP) has its roots in the 1970s, focusing on enabling computers to understand and interact with human language. In healthcare, NLP techniques have been utilized to extract information from clinical narratives, electronic health records (EHRs), medical literature, and patient-generated data. NLP has facilitated information extraction, sentiment analysis, clinical coding, and the development of conversational agents for healthcare applications.
1980s	Image Analysis	Image analysis became a significant root of AI in healthcare in the 1980s. Computer vision and image processing techniques were applied to medical imaging modalities, such as X-rays, CT scans, MRIs, and pathology slides, enabling the automated interpretation, segmentation, and detection of abnormalities. AI algorithms have enhanced medical imaging analysis, aiding in early disease detection, diagnosis, and treatment planning in fields such as radiology and pathology.
1990s	Robotics	Robotics started making an impact on healthcare in the 1990s, combining AI with mechanical devices to perform various medical tasks. Robotic systems have been developed for surgical procedures, rehabilitation, assistive care, and remote telemedicine applications. By incorporating AI algorithms, robotic systems can enhance precision, dexterity, and automation in healthcare, leading to improved outcomes, reduced invasiveness, and increased accessibility to medical services.
1990s	Data Mining	Data mining, or knowledge discovery from databases, became a key root of AI in healthcare in the 1990s. With the growth of electronic health records and the accumulation of vast numbers of healthcare data, data-mining techniques were applied to uncover hidden patterns, relationships, and insights. Data mining has contributed to population health management, disease surveillance, predictive modeling, and the identification of risk factors in healthcare.
1990s	Decision Support Systems	Decision support systems (DSS) emerged as a root of AI in healthcare in the 1990s, aiming to assist healthcare professionals in making informed decisions. DSSs incorporate AI techniques, such as rule-based systems, probabilistic models, and machine learning, to provide evidence-based recommendations, clinical guidelines, and alerts. DSSs have facilitated diagnosis, treatment planning, medication management, and improved patient safety in healthcare settings.
2000s	Knowledge Representation	Knowledge representation, which involves capturing and organizing knowledge in a structured format, has been a fundamental root of AI in healthcare. Various knowledge-representation techniques, such as ontologies, semantic networks, and knowledge graphs, have been applied to represent medical knowledge, clinical guidelines, and domain-specific information.

**Table 2:** Representative Machine-Learning Models for Healthcare Applications.

Model	Description	Pros	Cons
Convolutional Neural Networks (CNNs)	CNNs are widely used for image-based tasks in healthcare, such as medical imaging analysis, including classification, segmentation, and detection. They leverage specialized layers to extract features from images and have achieved remarkable success in areas such as radiology and pathology.	- Excellent performance in image analysis tasks - Automatic feature extraction - Ability to handle complex image structures	- High computational requirements - Require large numbers of labeled training data - Limited interpretability
Recurrent Neural Networks (RNNs)	RNNs are suitable for sequential data analysis and have been applied in various healthcare tasks. They can capture dependencies over time, making them valuable for tasks, such as time-series analysis, patient monitoring, and natural language processing in electronic health records (EHRs).	- Ability to capture temporal dependencies - Effective for sequential and time-series data - Widely used in NLP applications	- Vulnerable to vanishing/exploding gradients - Difficulty in modeling long-term dependencies - High computational requirements
Neural Networks	Neural networks, including multi-layer perceptrons (MLPs), are versatile models used in healthcare. They are composed of interconnected layers of artificial neurons, enabling them to learn complex patterns in both structured and unstructured healthcare data. They have been applied to various tasks, including disease diagnosis, risk prediction, and patient outcome analysis.	- Ability to learn complex patterns from data - Suitable for a wide range of healthcare tasks - Effective for both structured and unstructured data	- Require large numbers of labeled training data - Prone to overfitting - Interpretability can be challenging, especially for deep neural networks
Support Vector Machines (SVMs)	SVMs are a popular class of supervised learning algorithms used in healthcare. They are effective for classification tasks and have been applied in various areas, including disease diagnosis, risk prediction, and outcome analysis, by mapping data into high-dimensional feature spaces.	- Effective for high-dimensional data - Good generalization performance - Robust to overfitting	- Computationally expensive for large datasets - Require careful selection of the kernel function and hyperparameters - Lack probabilistic outputs
Random Forests	Random forests are an ensemble learning method that combines multiple decision trees to make predictions. They are versatile and have been used in disease diagnosis, prognosis, and feature selection by leveraging their ability to handle high-dimensional data and identify important features.	- Good performance for high-dimensional data - Ability to handle missing values and outliers - Provide feature importance ranking	- Can be slow for large datasets - Lack interpretability for individual trees
Deep Belief Networks (DBNs)	DBNs are generative models that employ unsupervised learning to learn hierarchical representations of data. They have shown promise in healthcare tasks, such as genetic analysis and medical imaging, and in clinical decision support systems by capturing complex patterns in large datasets.	- Ability to capture hierarchical representations - Effective for unsupervised feature learning - Suitable for large-scale datasets	- Computationally expensive for training - Require large numbers of labeled data for supervised fine-tuning - Difficult to interpret and understand the learned representations
Natural Language Processing (NLP)	NLP techniques are used to process and analyze human language data. They involve various tasks, such as sentiment analysis, text classification, named entity recognition, machine translation, and question-answering systems, enabling the understanding and extraction of information from textual data.	- Extraction of insights from unstructured textual data - Sentiment analysis and text classification - Named entity recognition - Machine translation for cross-lingual communication - Question-answering systems for information retrieval	- Ambiguity and context in natural language - Language complexity and variation - Lack of domain-specific data - Privacy and ethical concerns - Bias and fairness - Interpretability challenges
Decision Trees	Decision trees are simple yet powerful models used for classification and regression tasks. They partition data based on features to form a tree-like structure and make predictions. Decision trees are interpretable and can handle both categorical and numerical data.	- Easy to interpret and visualize - Can handle both categorical and numerical features - Nonlinear relationships between features can be captured	- Prone to overfitting, especially with complex trees - Sensitive to small variations in data

**Table 3:** Potential Applications of AI in Spinal Disease Care.

Area of Spinal Disease Care	Description
Diagnosis and Detection	AI can assist in the automated analysis of medical imaging data, such as MRI or CT scans, for the detection and segmentation of spinal conditions, such as spinal stenosis. AI algorithms can aid in accurate and efficient diagnosis, providing valuable insights for healthcare professionals.
Treatment Planning	AI can support healthcare professionals in personalized treatment planning for spinal diseases. By analyzing patient data, including medical images, clinical records, and outcomes, AI algorithms can help determine the most appropriate treatment options and assist in surgical technique selection.
Surgical Guidance	AI can provide real-time guidance during spinal surgeries. By integrating pre-operative imaging data and intraoperative feedback, AI systems can help surgeons navigate complex spinal anatomies and make informed decisions, leading to improved surgical outcomes.
Predictive Modeling	AI can develop predictive models to assess disease progression and treatment outcomes for spinal diseases. These models can aid in prognosticating patient outcomes, optimizing treatment strategies, and facilitating shared decision making between healthcare providers and patients.
Rehabilitation Support	AI can assist in designing personalized rehabilitation programs for patients with spinal diseases. By analyzing patient data, including movement patterns and sensor data, AI algorithms can provide customized recommendations and monitoring during the rehabilitation process.
Remote Monitoring	AI-enabled remote monitoring systems can help track and monitor patients with spinal diseases outside of healthcare facilities. These systems can provide continuous monitoring, detect changes in symptoms or movement patterns, and alert healthcare providers for timely intervention.

**Table 4:** Limitations of and improvement measures for AI and ML in spinal care.

Limitations	Improvement Measures
Lack of high-quality, diverse data	Ensure data collection with proper annotation and labeling Expand data sources and collaborations Implement data augmentation techniques to increase dataset diversity
Potential bias and fairness issues	Develop bias detection and mitigation techniques Implement fairness-aware algorithms Conduct rigorous evaluation of models for bias and fairness
Interpretability and explainability	Develop transparent and interpretable AI and ML models Create model-agnostic interpretability techniques Provide explanations for model predictions and decisions
Generalizability and transferability	Collect data from diverse populations and settings Explore transfer-learning and domain-adaptation techniques Conduct external validation studies across different healthcare settings

## Data Availability

Data sharing not applicable. No new data were created or analyzed in this study.
